# Immune modulation of hair follicle regeneration

**DOI:** 10.1038/s41536-020-0095-2

**Published:** 2020-05-11

**Authors:** Waleed Rahmani, Sarthak Sinha, Jeff Biernaskie

**Affiliations:** 1 Department of Medicine, Cumming School of Medicine, University of Calgary, Calgary, AB T2N 1N4 Canada; 2 Department of Comparative Biology and Experimental Medicine, Faculty of Veterinary Medicine, University of Calgary, Calgary, AB T2N 1N4 Canada; 3 Hotchkiss Brain Institute, University of Calgary, Calgary, AB T2N 1N4 Canada; 4 Alberta Children’s Hospital Research Institute, University of Calgary, Calgary, AB T2N 1N4 Canada

**Keywords:** Skin stem cells, Autoimmunity

## Abstract

The mammalian hair follicle undergoes repeated bouts of regeneration orchestrated by a variety of hair follicle stem cells. The last decade has witnessed the emergence of the immune niche as a key regulator of stem cell behavior and hair follicle regeneration. Hair follicles chemotactically attract macrophages and T cells so that they are in range to regulate epithelial stem cell quiescence, proliferation and differentiation during physiologic and injured states. Disruption of this dynamic relationship leads to clinically significant forms of hair loss including scarring and non-scarring alopecias. In this review, we summarize key concepts behind immune-mediated hair regeneration, highlight gaps in the literature and discuss the therapeutic potential of exploiting this relationship for treating various immune-mediated alopecias.

## Introduction

Hair follicles (HF) serve a wide range of functions including thermoregulation, physical protection, sensory input, and decorative purposes for social interactions. Exposure to the environment has conferred a remarkable capacity for episodic regeneration under numerous conditions including wounding, plucking, pregnancy, and after the application of select cytokines and immunomodulatory drugs^1^. To support the cellular demand of life-long regeneration, HFs rely on an assortment of stem cells located in distinct anatomical regions to supply differentiated progeny. Collectively known as hair follicle stem cells (HFSCs), they include slow-cycle epithelial stem cells of the bulge^2^, fast-cycling epithelial progenitors in the secondary hair germ^3^, dermal stem cells of the lower dermal sheath^4,5^, and melanocyte stem cells^6^. HFSC behavior is tightly regulated by extrafollicular signals as evidenced by the fact that hair regeneration occurs within discrete boundaries implying coordination among adjacent follicles and the extrafollicular environment^7^. Various sources of extrafollicular signals have been discovered including intradermal adipocytes^8^, dermal fibroblasts^7^, blood vessels^9^, lymphatic vessels^10^ and peripheral nerves^11^. Characterizing the dynamic signaling networks within this niche is critical for understanding how stem cells are mobilized to self-renew, differentiate, maintain tissue homeostasis and how their dysfunction contributes to cutaneous disorders.

The immune system maintains organismal harmony beyond the traditional cellular and humoral defense mechanisms discovered over a century ago. Throughout the body, the immune system generates discrete milieus permissive for proliferation, differentiation, quiescence, and extra-cellular matrix deposition in order to support local regenerative efforts. Pro-regenerative mechanisms include debris and senescent cell clearance, angiogenesis and the modulation of immune cell heterogeneity^12–18^. Another strategy employed by immune cells is to call upon lineage restricted stem cells to provide differentiated cells necessary for functional tissue^17,19–26^. For example, FoxP3^+^CD4^+^ regulatory T (T_reg_) cells directly act upon satellite stem cells of the skeletal muscle through amphiregulin to promote muscle repair after injury^27^. Another immune-mediated strategy, admittedly a less well-understood one, is cellular de-differentiation and reprogramming toward a plastic embryonic-like state after injury as witnessed during blastema-formation during axolotl limb regeneration^28^. Early inflammatory signals from macrophages are indispensable to induce downstream changes in the wound epithelium for proper blastema formation^29^.

Discoveries in the mid-20th century gave birth to the field of trichoimmunology, the study of the HF immune system, and spurred investigations into the mechanisms underlying immune privilege^30–33^, immune-mediated alopecia^34–38^, and maintenance of immune tolerance^39–41^. Classic allograft experiments by Billingham and Silvers showed that the epithelial hair bulb ranks among the few immunologically privileged sites of the mammalian body^42^. Black hair shafts from skin transplanted onto white guinea pig pierced the skin and persisted for up to 100 days post transplantation. Early ultrastructural analyses also revealed that the bulk of normal skin flora reside in HF openings. Considering the rarity of folliculitis, HFs must be critical mediators of immune tolerance against commensal microbes^43^. Indeed, recent findings have established HFs as indispensable portals for immune cells into the cutaneous niche^40^. In the last decade, trichoimmunology has expanded to include the study of immune-mediated hair regeneration through the direct manipulation of HFSCs and de-differentiation of dermal and epidermal wound cells. A glut of new studies revealed that T cells and macrophages are the most promiscuous immune regulators of HF regeneration during physiologic hair cycling^44,45^, injury-induced regeneration^46–50^, and injury-induced hair neogenesis^51^. Disrupting or exaggerating this relationship can lead to clinically significant forms of immune-mediated alopecia ^35,38,52^.

This review will begin by briefly summarizing the various immune cells resident within the skin followed by a brief overview of the mechanisms underlying HF regeneration. We will then examine how the immune niche modulates HF regeneration under physiologic and injured states followed by a discussion regarding the clinical and therapeutic implications of these findings. Throughout our discussion we will highlight outstanding questions within the literature.

### The cutaneous immune system

**Fig. 1:**
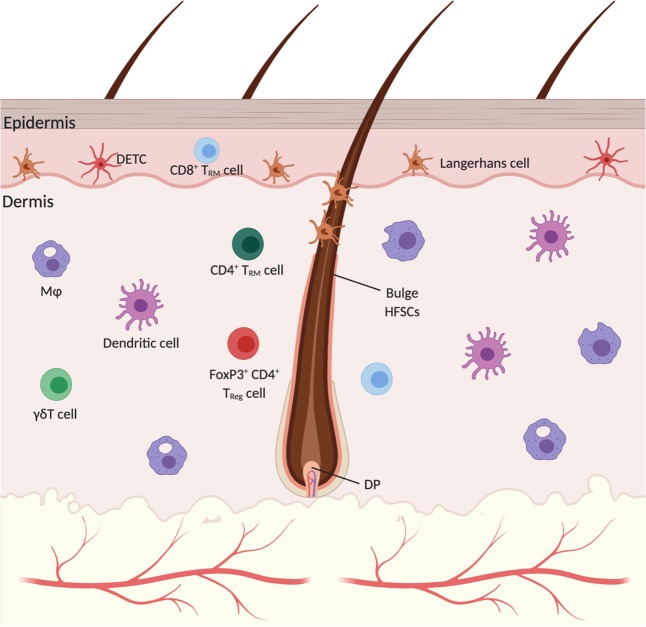
An overview of the murine cutaneous immune system. The epidermis is primarily home to Langerhans cells and dendritic epidermal T-cells (DETC). CD8+ T cells are found sporadically within the epidermis as well. Within the dermis is a diverse collection of immune cells that vary in their prevalence depending on anatomical site, depth, proximity to HFs and HF stage. Macrophages and dendritic cells are the dominant immune cells of the murine dermis. CD4^+^ T resident memory (T_RM_) and regulatory (T_reg_) cells are found alongside CD8^+^ T_RM_ and γδT cells. FoxP3^+^ T_reg_ cells are spatially biased to the peri-follicular space. DP—dermal papilla. Created with BioRender.com.

A diverse collection of immune cells are interspersed throughout the skin (Fig. 1)^53^. Mononuclear phagocytes are the most abundant dermal leukocytes particularly dendritic cells and macrophages. Both are found decorating the superficial dermis whereas the deeper reticular regions of the dermis are largely dominated by macrophages^54^. Macrophages are traditionally dichotomized into M1 and M2 subtypes, pro-inflammatory and anti-inflammatory, respectively. However, this binary classification is a relic of the pre-genomic era and grossly over simplifies their phenotypic and functional diversity^55^. They are capable of dynamically and reversibly responding to environmental stimuli acquiring new functional properties beyond their classical inflammatory functions including tissue repair^56–59^. Flow analyses of the steady-state murine dermis using CD64 and CCR2 revealed six distinct mononuclear phagocyte populations: CD11b+ dendritic cells, monocytes, two monocyte-derived dendritic cell populations (CD64^lo^ and CD64^+^), and two distinct macrophage populations (MHCII^+^ and MHCII^−^)^60^. The six subpopulations had varying capacities for T cell stimulation, antigen presentation, migration, and scavenging^60^. Langerhans cells (LC’s), dendritic cells of the epidermis, make up approximately 2–3% of epidermal cells and constitute the first immunologic barrier to the external environment. They are professional antigen presenting cells that survey foreign antigens through extended dendrites and migrate to regional cutaneous lymph nodes^61^. In human HFs, LCs are also found in the infundibular epithelium, follicular bulge and sebaceous epithelium ^30,62^.

Non-inflamed adult human skin is home to approximately 20 billion T lymphocytes^63^. The two broad classes of cutaneous T cells include γδT cells and αβT cells. Much of what we know regarding the in vivo functions of cutaneous γδT cells comes from γδT cell receptor deficient (TCRδ^−/−^) mice. Epidermal γδT cells, also known as dendritic epidermal T cells (DETCs), are found in murine but not human skin, express a conserved Vγ5 Vδ1 T cell antigen receptor and are distinguished from “conventional” lymphocytes by their similarity with innate immune cells as cytokines secretors during the early stages of inflammation and tissue dysregulation^64,65^. DETCs form tight associations with neighboring keratinocytes through E-cadherin, remain largely immobile, and appear to be critical for epidermal homeostasis and repair^66^. Indeed, wounds show impaired keratinocyte proliferation, diminished inflammation, defects in macrophage recruitment, and slower closure in the absence of DETCs^67^. Dermal γδT cells, on the other hand, comprise nearly 50% of the total dermal T cell population, are found in humans, are migratory, and are thought to be involved in pathogen defense by augmenting neutrophil recruitment through IL-17^68^. αβT cells are broadly divided into CD4^+^, CD8^+^, and natural killer (NK) T cells. The vast majority are tissue resident memory T (T_RM_) cells that provide local protection against infection independent from the circulatory pool^69^. Both CD4^+^ and CD8^+^ T cells are observed within in the follicular epithelium, however only CD8^+^ T cells are found in the interfollicular epidermis^70^. About 10% of human cutaneous CD4^+^ cells are immunosuppressive FoxP3^+^ T_reg_ cells that function to relieve inflammatory skin disorders like atopic dermatitis, contact hypersensitivity and psoriasis ^71–73^.

Interestingly, the composition of immunocytes in the perifollicular space is distinct from the surrounding interfollicular space. Mast cells, macrophages, LCs, and T cells preferentially associate to the HF in murine and human skin^30,74^. This is loosely analogous to the mucosal-associated lymphoid structures seen within many of the submucosal membrane sites of the body including the gastrointestinal tract, breast, lung and nasopharynx. In fact, disruption of the HF-associated lymphatic network, conduits for local immune responses and macromolecule drainage, led to precocious HF cycling highlighting their role in HFSC quiesence^10,75,76^. Through a rich array of cytokines, HF-associated immune cells ward off pathogens, prevent dysregulated immune activity and support tissue homeostasis by fine-tuning HFSC behavior.

### Hair follicle stem cells: hair cycle conductors

To understand how immune cells modulate HF regeneration, a brief introduction into the cellular and molecular cascades controlling the hair cycle is warranted. HFs cycle through three different phases: telogen (relative quiescence), anagen (growth), and catagen (degeneration). Multiple stem cell and progenitor populations in distinct anatomical sites are responsible for coordinating the proliferation, differentiation, and migratory behaviors necessary for continuous regeneration. In 1859, the German histologist Franz von Leydig was first to describe the HF bulge of murine vibrissae as “…a thickening or a circular bulge…located on the upper third of the hair root…with only the outer root sheath contributing to this feature”^77^. The significance of this structure was not appreciated until 1990 when a subpopulation of bulge keratinocytes were discovered to be slow-cycling and label retaining cells^2^. Today, these keratinocytes are understood to be stem cells capable of generating all epithelial compartments of the HF and the interfollicular epidermis after wounding^78–82^. Key bulge stem cell markers include CD34, Lgr5, Sox9, NFATc1, Tcf3, keratin 15, Lgr6, and Lhx2^79,80,83–87^. Fast-cycling P-cadherin^+^ progenitors are found in the secondary hair germ, the compartment sandwiched between the bulge and dermal papilla^88^. Cells of the hair germ are transcriptionally more active than bulge cells and begin to proliferate in late telogen, a few days before bulge cells^3^. The mesenchymal compartment is comprised of the dermal sheath and the dermal papilla, a cluster of inductive cells at the base of the follicle. Both populations are maintained by αSMA^+^Sox2^+^ dermal stem/progenitor cells within the lower dermal sheath named the hair follicle dermal stem cell (hfDSC)^4,5,89^. Modern laser and genetic ablation approaches confirmed early microdissection experiments that the follicle is stunted in telogen without the dermal papilla ^89–93^.

HFs are held in telogen due to elevated BMP signaling in quiescent HFSCs^80,94^. To enter the regenerative phase of the hair cycle, reversal of BMP-mediated quiescence and activation of the epithelial differentiation program through WNT signaling is required^3,7,94–96^. In late telogen, BMP signaling within the hair germ is lost due to the expression of BMP inhibitors Noggin, Bambi, TGFβ2 and Sostdc1 by the dermal papilla^3,95,97,98^. Noggin knockout mice show a significantly reduced number of anagen follicles. Intradermal injections of Noggin is sufficient to induce anagen in telogen skin^97,98^. Once BMP-mediated repression is lifted, stabilization of nuclear β-catenin, mediated by continued contact with the dermal papilla, becomes necessary to activate hair germ cells and promote their conversion to proliferating transit-amplifying cells^3,85,99–102^. Hair germ-derived sonic hedgehog (SHH) is then sensed by bulge HFSCs to self-renew and form the outer root sheath^103,104^. SHH also instructs the dermal papilla to potentiate hair germ proliferation and lineage differentiation through the secretion of FGF7 and 10 ^3,103^.

The molecular interplay between the dermal papilla, hair germ, and bulge is central to the episodic nature of HF regeneration. It is important to appreciate that this regenerative network is not autonomous but highly sensitive to cutaneous and systemic signals. The following sections will describe how immune-derived signals participate in HF regeneration by co-opting the aforementioned regenerative programs.

### Immune-mediated physiological hair follicle cycling

**Fig. 2:**
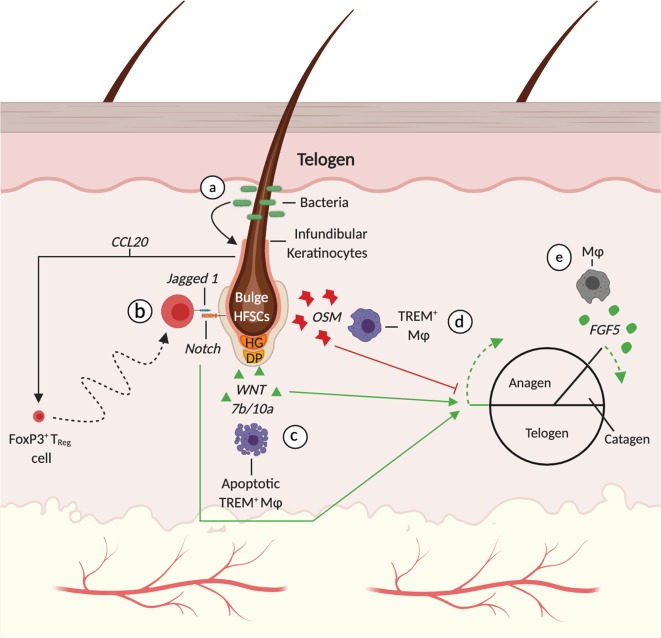
Immune-mediated modulation of homeostatic HF regeneration. HFs undergo a continuous cycle of regeneration and degeneration defined by three distinct phases: telogen, anagen and catagen. **a** FoxP3^+^ T_reg_ cells are recruited to the peri-follicular space by CCL20-secreting infundibular keratinocytes augmented by commensal microbes. **b** FoxP3^+^ T_reg_ cells activate the proliferation and differentiation programs of Lgr5+ HFSCs through Jagged 1 and Notch signaling. **c** HFSC quiescence is released after TREM^+^ macrophages apoptosis and OSM levels decline. Anagen induction begins with the release of Wnt7b and 10a from apoptotic macrophages. **d** TREM^+^ macrophages maintain HFSC quiescence and telogen by upregulating JAK-STAT5 signaling through Oncostatin M (OSM). **e** Macrophages also contribute the end stages of anagen as they promote catagen and degeneration through Fgf5 secretion. DP Dermal papilla, HG hair germ, HFSCs hair follicle stem cells. Created with BioRender.com.

Murine hair cycling occurs in regenerative waves characterized by dramatic architectural and mitotic changes across the entire skin. Early observational studies revealed significant fluctuations in immune cell numbers that correlated with hair cycling^74,105–109^. Broadly speaking, the perifollicular numbers of CD4^+^ and CD8^+^ T cells, dermal γδT cells, and macrophages reach their nadir in telogen before peaking during mature stages of anagen^107^. On the other hand, CD4^+^FoxP3^+^ T_reg_ cells and mast cells decline dramatically during anagen while intrafollicular LC’s and γδT cell numbers remain unchanged during the hair cycle^44,107,109^. For decades, evidence of a functional relationship between the synchronized remodeling of the cutaneous immune system and HF has been wanting. It was not clear whether the variations in immune cell numbers were a consequence of hair cycling or vice versa. Compelling evidence has emerged painting a complex relationship defined by mutual responsibility between HFs and immune cells during physiologic hair regeneration with T cells and macrophages as the predominant actors (Fig. 2).

The earliest evidence for a link between hair-cycle associated fluctuations in cutaneous immune cells and hair regeneration was the observation that allograft rejection time is longer when the HFs in donor skin are in telogen as opposed to anagen^110^. Early ultrastructural images of catagen follicles showed macrophages engulfing and degrading collagen fibers of the connective tissue sheath. The implication of these findings is that HF degeneration is predicated on the anagen-associated rise of macrophage numbers whom in turn phagocytize cellular debris during catagen^111,112^. There remains, however, no convincing data for the modulation of HF cycling through phagocytosis. Rather, macrophages owe their influence on the HF cycle to their impressive cytokine secretome particularly during the telogen-to-anagen transition. The JAK-STAT pathway, paralleling BMP-mediated quiescence, maintains HFSCs in relative quiescence during telogen^113^. Using single-cell RNA (scRNA) sequencing, genetic ablation models and hair reconstitution assays, TREM^+^ macrophages were shown to potentiate JAK-STAT mediated repression of HFSC proliferation through Oncostatin M (OSM)^114^. HFSC activation and differentiation occurred only after an apoptotic-driven decline in OSM secreting macrophages in late telogen^45,114^. Clodronate-mediated apoptosis of macrophages during telogen led to the release of Wnt7b and Wnt10a, activation of β-catenin/Wnt signaling in HFSCs and premature anagen entry (Fig. 2c, d)^45^. Interestingly, pharmacologic modulation of toll-like receptors have corroborated these findings. Topical application of imiquimod activated HFSCs and initiated premature anagen by reducing the number of inhibitory resident macrophages and increasing the number of activating infiltrating cells in late telogen^115^. Altogether, tissue-resident macrophages sustain telogen by upregulating quiescent transcriptional networks until their programmed cell-death in late telogen. Questions persist regarding the mechanisms orchestrating macrophage apoptosis in late telogen and the relationship between circulating monocytes and hair-regenerative macrophages. Outside of the telogen-to-anagen transition, macrophages promote catagen progression through FGF-5 (Fig. 2e)^116,117^.

Far from being passive recipients of immune-derived signals, HFs recruit immune cells to the parafollicular space to establish an immunologic niche favorable for regeneration. HF-derived IL-7 and IL-15 is necessary for the maintenance of cutaneous CD8^+^ and CD4^+^ memory T cell populations^70^. During the neonatal period and onset of bacterial colonization, FoxP3^+^ T_reg_ cells rapidly accumulate in the parafollicular space where they are trained to mount responses proportionate to the pathogenic insult without targeting self-antigens and commensal microbes^118^. Because T_reg_ cells reside in close association to human and mouse HFs, reason follows that follicle-derived signals are responsible for their rapid accumulation^41,118,119^. Indeed, abrogation of HF morphogenesis by overexpression of the Dkk1, a WNT inhibitor, in K5^+^ epithelial cells significantly reduced the number of T_reg_ cells in postnatal day 13 skin^39^. Moreover, germfree mice exhibited a similar phenomenon suggesting that bacterial colonization is necessary to recruit cutaneous T_reg_ cells. qRT-PCR chemokine arrays revealed that commensal microbes augment HF infundibular keratinocyte secretion of CCL20 to promote neonatal T_reg_ cell recruitment (Fig. 2a)^39^. Ablation studies exposed a role for T_reg_ cells in natural hair cycling. FoxP3^DTR^ mice treated with diphtheria toxin (DT) at the first telogen-to-anagen transition lacked CD4^+^ CD25^+^ FoxP3^+^ T_reg_ cells, presented CD34^+^ HFSCs with decreased proliferative capacity, and failed to enter anagen^44^. T_reg_ cells stimulate HFSC differentiation and proliferation through direct cell-cell interactions via the Jagged 1 (Jag1)-Notch pathway. Injection of Jag1-Fc coated microbeads or cre-mediated excision of *jag1* in FoxP3^cre^ × Jag1^fl/fl^ mice significantly attenuates bulge HFSC proliferation and depilation-induced regeneration (Fig. 2b)^44^. T_reg_ cells critical role in hair regeneration is clinically illustrated in the IPEX (immune dysregulation, polyendocrinopathy, enteropathy X-linked) syndrome, a rare genetic disorder caused by a mutation in the human FOXP3 gene causing aberrant T_reg_ cell development and systemic autoimmune dysfunction including alopecia universalis, a severe form of hair loss characterized by the loss of all hair^52,120^.

Together, tissue-resident macrophages and T_reg_ cells coordinate physiologic HF regeneration by directly manipulating HFSC behavior through native stem cell activation and differentiation programs including JAK-STAT, β-catenin/Wnt, and Jag1-Notch signaling. T cells go further by establishing an environment conducive for cyclical regeneration through coordinated efforts with commensal microbes. Are macrophages similarly impacted by the cutaneous microbiome? Moreover, it is unclear whether macrophages and T cells cooperate to exert molecular influence upon the HF cycle. Synergy between the innate and adaptive immune system is central for a well-functioning defense system and whether this extends into physiologic regeneration remains to be seen.

### Injury-induced regeneration

**Fig. 3:**
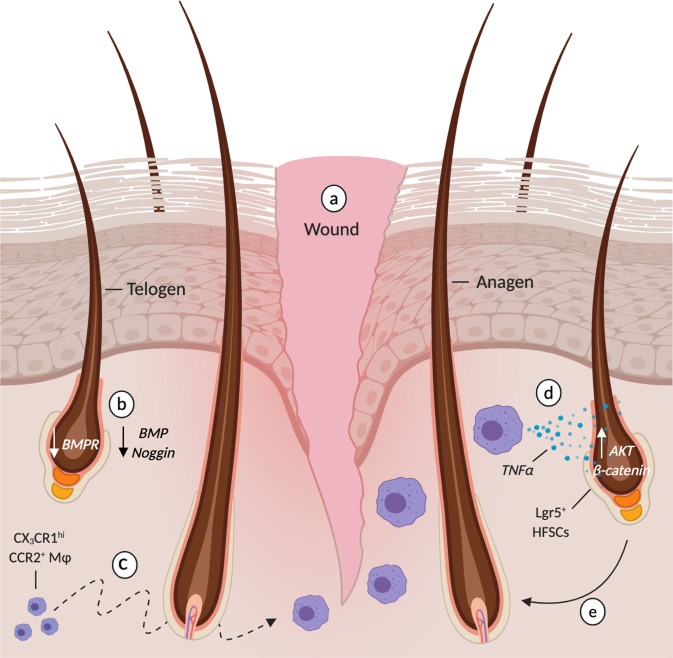
Immune-mediated hair regeneration during wound-induced hair growth. **a** Full thickness wounds stimulate circumscribing telogen follicles into anagen. **b** After injury, BMP-mediated repression of HFSC activity is alleviated as perifollicular levels of BMP and noggin decline. **c** Perifollicular concentrations of CX_3_CR1^+^ CCR2^+^ wound macrophage rise 7–11 days after injury in a CX_3_CR1 and TGFβ1 dependent fashion. **d** CX_3_CR1^+^ CCR2^+^ macrophage-derived TNFα upregulates AKT/β-catenin in Lgr5^+^ HFSC’s no longer repressed via BMP. **e** Activated Lgr5+ HFSCs proliferate and differentiated into the keratinocytes necessary for anagen transition and the generation of a hair fiber. Created with BioRender.com.

**Fig. 4:**
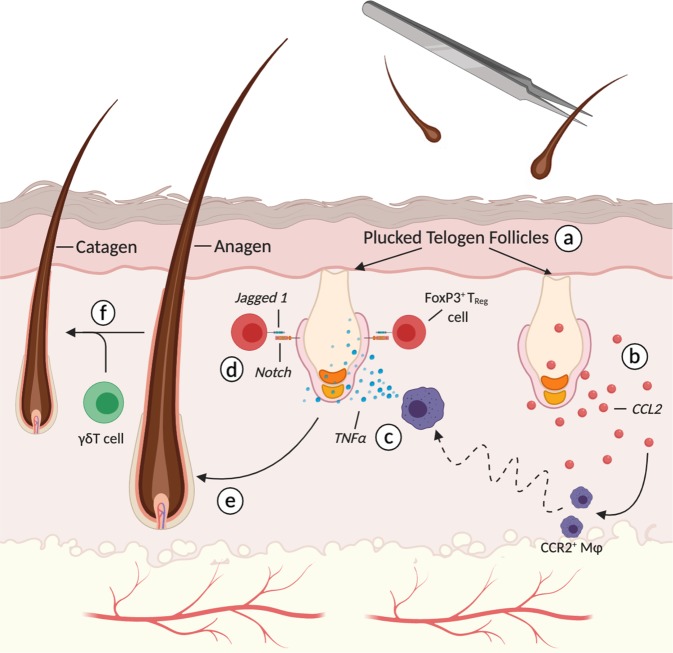
Immune-mediated hair regeneration during depilation-induced hair growth. **a** Depilated telogen HFs, via plucking, are induced to regenerated including neighboring unplucked follicles. **b** Keratinocytes from plucked follicles secrete CCL2, a chemotactic signal responsible for recruiting CCR2^+^ macrophage to the perifollicular space. **c** TNFα from recruited macrophage is necessary for HFSC activation. **d** Concomitantly, FoxP3^+^ T_reg_ cells activate the proliferation and differentiation programs of Lgr5+ HFSCs through Jagged 1 and Notch signaling. **e** Activation of HFSCs directly leads to anagen growth in plucked HFs. **f** Through unknown molecular mechanisms, γδT cells control the progression of anagen to catagen. Created with BioRender.com.

The wound healing literature has offered valuable insights into immune-mediated hair regeneration. The preferred models for studying injury-induced hair regeneration have been wound-induced hair growth (WIHG) (Fig. 3) and depilation-induced regeneration (Fig. 4). In each case, injury stimulates regenerative waves in the surrounding skin as circumscribing telogen follicles are activated into anagen. The first inquiries into the biological mechanisms underlying WIHG examined whether the release of an activating substance or the loss of an inhibitor was responsible for hair growth^121,122^. Argyris and Trimble approached this by asking whether the removal of a cutaneous tumor mass was sufficient to stimulate WIHG. They concluded that neither the loss of an inhibitor nor the release of an activating substance was sufficient to initiate WIHG. Furthermore, they hypothesized that the competence of telogen follicles must explain the wide variations in rate, amount and pattern of hair growth stimulation^121^. Fifty years later, the relative expression of BMP and Wnt/β-catenin was discovered as one of the molecular determinants responsible for regenerative competence of telogen follicles^7^. Indeed, wounding tips the balance towards regenerative competence by downregulating cutaneous and follicular BMP-mediated repression^123^. As for the activating signal, macrophages and T cells have emerged as potent stimulators of HFSC differentiation and proliferation.

The first indication that macrophages are required for injury-induced hair regeneration came from the observation that ASK1^−/−^ wounds lacked infiltrating F4/80^+^ macrophages and exhibited significantly delayed WIHG^49^. The causal link was made after intradermal transplantation of bone-marrow derived macrophages rescued hair growth in ASK1^−/−^ wounds^49^. However, many questions remain unanswered. How are macrophages recruited to the site of injury? Is there a specific macrophage phenotype and secreted factor responsible for stimulating injury-induced regeneration? How does the macrophage-derived signal alter HFSC behavior and promote regeneration? Multiple research groups, including ours, shed light on the molecular pathways responsible for macrophage-mediated hair regeneration after injury. Using depilation as a model for microinjury, Chen and colleagues described an elegant two-step mechanism responsible for plucking-induced regeneration^47^. Through mathematical modeling, they approximated the putative signal’s decay length to 1 mm, an order of magnitude larger than that of diffusible receptor-binding ligands, and hypothesized a role for recruited cells. Indeed, experimental data revealed that pro-inflammatory macrophages are recruited by plucked follicles via CCL2 to regenerate plucked and unplucked follicles through TNFα (Fig. 4b, c)^47^. Using the CX_3_CR1^gfp/−^:CCR2^rfp/−^ transgenic mouse, our group showed that anti-inflammatory CCR2^+^CX_3_CR1^hi^Ly6c^lo^ TNFα^+^ macrophages are required for WIHG in a TGFβ1 and CX_3_CR1 dependent manner; signals crucial for chemotaxis and survival after extravasation, respectively^46,124–127^. Microarray and proteomic analysis of infiltrating macrophages revealed that macrophage-derived TNFα stimulates hair regeneration by activating AKT/β-catenin signaling in Lgr5^+^ HFSCs (Fig. 4c)^48^.

As with homeostatic regeneration, T cells manipulate HFSC behavior during injury-induced regeneration through Jag1-Notch signaling (Fig. 4d)^44^. How T_reg_ cells are activated by injury to stimulate HFSC proliferation remains unknown. Does the stimulus originate from the follicle, similar to CCL2-mediated recruitment of macrophages, or does it come from surrounding damaged tissue? Moreover, the dramatic effect of individually depleting T_reg_ cells and macrophages on injury-induced regeneration suggests that Jag1 and TNFα are not redundant regenerative signals. Instead, these two immune-derived signals may behave synergistically as they promote HF regeneration after injury.

So far, we have only considered HFSCs role in regenerating the epithelial compartments of anagen follicles. Supplying differentiated progeny to the interfollicular epidermis after epidermal injury is a secondary function of these epithelial stem cells. Indeed, hair-bearing skin heals more rapidly than non-hairy skin^82,128^. Modeling subacute epidermal injury with repeated applications of adhesive tape while lineage tracing Lgr5^+^ bulge cells in T_reg_ cell depleted mice revealed that the HFSC differentiation program is directed toward restoring skin-barrier integrity in a T_reg_ cell dependent fashion^129^. Lgr5^+^ cells from T_reg_-deficient mice expressed higher levels of bulge-associated genes (*cd34*, *cd200*, and *postn*) as opposed to the terminally differentiated keratinized gene set (*keratin 1* and *involucrin*) seen in wildtype mice. T_reg_ cells indirectly skew the HFSC differentiation program and migration pattern by attenuating the neutrophil-associated CXCL5-IL17 inflammation axis. Neutralization of CXCL15 and IL17 restored epidermal repair in T_reg_ depleted mice^129^. However, it is unclear how Lgr5^+^ HFSCs are directly influenced by this complex inflammatory axis. Another signaling axis capable of directing HFSC differentiation towards the interfollicular epidermis is IL-1 and γδT cells^50^. Wounding upregulates epidermal-derived IL-1α, a potent activator of γδT cells whom in turn activate HFSC proliferation and migration. γδT cells also control the latter stages of injury-induced hair regeneration. Anagen induction after depilation was comparable in wildtype and mice lacking γδT cells (TCRδ^−/−^ mice) while the onset of follicle regression (catagen) was markedly delayed in TCRδ^−/−^ mice (Fig. 4f) ^130^.

Fifty years after Arygris and Trimble hypothesized that injury-induced regeneration is dependent upon follicular competence via the loss of an inhibitor and presence of a molecular activator, macrophages and T cells have emerged as critical mediators of the latter. However, it is unclear what the relative contribution of circulating monocytes and resident macrophages is during injury-induced regeneration. Unlike microglia of the CNS and Kupffer cells of the liver, dermal macrophages are maintained by circulating monocytes during adulthood, therefore it is reasonable to surmise that injury-induced regeneration is a systemic response to trauma originating in primary lymphoid organs such as the bone marrow and spleen. Future parabiotic and skin allograft studies would help address whether the local immune niche is sufficient or even necessary for injury-induced hair regeneration. Moreover, the impact of aging or metabolic diseases like diabetes on immune niche and regeneration is unclear. Finally, future endeavors will be served well to study macrophages and T cells concomitantly with the aid of next generation single cell transcriptomic tools and datasets.

### Injury-induced neogenesis

**Fig. 5:**
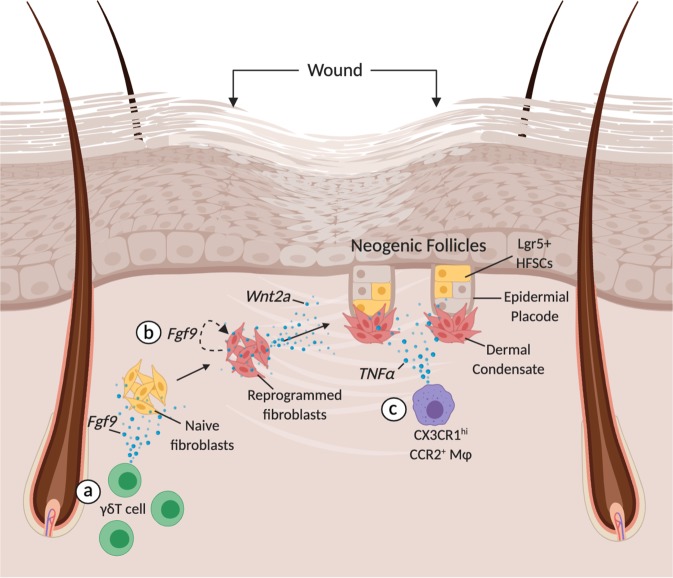
Immune-mediated cellular reprogramming and hair neogenesis. Large full-thickness wounds on murine backskin stimulate de novo folliculogenesis in the wound center. **a** γδT cells are actively recruited where they reprogram naïve dermal fibroblasts through Fgf9. **b** A cascade of signaling pathways ensue where the reprogrammed dermal fibroblasts sustain their activated state through autocrine Fgf9 signaling followed by dermal Wnt secretion. Reprogrammed dermal fibroblasts are precursors to the dermal condensate and dermal papilla. **c** TNFα from CX3CR1^+^CCR2^+^ macrophages is also necessary for proper HF neogenesis. TNFα promotes PI3K/AKT signaling in Lgr5+ cells of the nascent epidermal placode. Created with BioRender.com.

In the 1950s, Charles Breedis generated pure squamous sheets of rabbit epithelium by forcing large wounds to heal without contraction and to his surprise he observed functioning HFs and sebaceous glands within the scar^131^. This contradicted dogma that HFs only develop during late embryogenesis and the loss of adult follicles is permanent. Therefore, de novo HF formation was disregarded for half a century even as the phenomenon was reproduced in mice and humans^132,133^. The last decade has seen a resurgence of studies examining the molecular mechanisms underlying wound-induced hair neogenesis (WIHN) exposing another role for immune cells in hair regeneration (Fig. 5). Follicular neogenesis begins 12–19 days after inflicting a full-thickness wound measuring 1–2.25 cm^2^ with a final healing diameter of at least 0.5cm^134^. Excluding their disorganized orientation and lack of pigment, neogenic follicles are functionally indistinguishable from surrounding follicles as they pass through the entire hair cycle. On a molecular level, WIHN parallels the development of embryonic follicles with the expression of Krt17, Lef1, alkaline phosphatase, and Wnt10b^134^. Other critical pathways regulating hair neogenesis include Shh, Msx2, and IL-6^135–137^. The origin of the de novo bulge HFSCs and inductive dermal papilla remains unknown. Are they derived from lineage-restricted stem cells or do local cells expand their lineage plasticity in a blastema-like mechanism? Giving credence to the latter mechanism, Lim and colleagues showed that neogenic DP’s are derived from reprogrammed wound fibroblasts with access to developmental programs responsible for their regenerative fate^136^. Various epithelial lineage tracing experiments have failed to conclusively delineate the origin of neogenic bulge HFSCs although progeny of Lgr5^+^ and Lgr6+ but not Krt15^+^ HFSCs were found in neogenic follicles^48,134,138^. The impressive cellular plasticity during WIHN suggests a role for undescribed epigenetic changes driving the de-programming and re-programming cascades within epithelial and mesenchymal cells. What was once perceived a regenerative mechanism exclusive to early vertebrates, blastema-like regeneration has become a plausible method of mammalian regeneration, a developmental network buried beneath years of evolutionary history resurfacing in times of significant injury^139^.

Immune cells are critical for initiating the complex interplay between epithelial and mesenchymal cells during WIHN. One such signaling cascade is the activation and amplification of Wnt signaling between dermal fibroblasts and the epidermis^134,140^. Gay and colleagues showed that 10–12 days post wounding γδT cell-derived Fgf9 stimulated Wnt2a and Fgf9 expression in dermal fibroblasts (Fig. 5a, b)^51^. In fact, both TCRδ^−/−^ mice and mice lacking Fgf9 in T cells (Lck-Cre; Fgf9^fl/fl^) showed significant reduction in neogenic hairs, a deficiency that was not rescuable by Wnt7a overexpression in K14^+^ cells. Collectively, the authors proposed that dermal Wnt activation is stimulated by γδT cell-derived Fgf9, perpetuated by dermal Fgf9 in an autocrine fashion, precedes epidermal Wnt expression and is critical for WIHN^51^. It is unclear whether similar mechanisms are responsible for human WIHN. The low propensity for WIHN in human skin, known to harbor few γδT cells, suggests de novo hair neogenesis is strictly dependent on γδT cell-derived factors^51^.

A series of cellular and molecular cues resembling those responsible for macrophage-induced WIHG have been described for WIHN^48^. After injury, macrophage-derived TNFα activates AKT/PI3K signaling in Lgr5^+^ cells of the re-epithelized epidermis thereby encouraging folliculogenesis (Fig. 5d). DT-mediated ablation of LysM^+^ myeloid-derived macrophages during the early phases of injury abolished WIHN indicating that blood-derived and not tissue resident macrophages are required. Considering macrophages remarkable plasticity within the wound bed, it is difficult to interpret which phenotype is responsible for inducing neogenesis in the later stages of injury^46,141^. Likely, infiltrating Ly6C^hi^ CCR2^+^ CX_3_CR1^lo^ inflammatory macrophages transition into a Ly6C^lo^ CCR2^+^ CX_3_CR1^hi^ reparative phenotype before stimulating hair neogenesis. A histomorphometric analysis comparing the macrophage phenotypes in neogenic and non-neogenic domains along with phenotype specific depletion experiments will help to clarify the inductive phenotype. Wounds with TNF-deficient macrophages or from TNF^−/−^ mice exhibited fewer neogenic follicles compared to wild-type mice. Moreover, cre-mediated excision of Pten, the negative regulator of the AKT/PI3K pathway, in Lgr5^+^ HFSCs increased the number of neogenic follicles in the wound. Altogether, Ly6C^+^ macrophages induce Lgr5^+^ HFSC activation and hair neogenesis through TNFα in a AKT/PI3K dependent fashion. The temporal relationship between macrophage and γδT cell mediated regeneration through TNFα and FGF9, respectively, is unclear.

Neogenic follicles have a curious predilection for the wound center forming in clusters surrounded by hairless scar suggesting a bias for inductive signals and cellular reprogramming to the wound center. Indeed, TNF-luciferase expression shifted from the wound periphery to the center 10 days post injury^48^. This central tendency might be explained by a reaction-diffusion model similar to the WNT/DKK model where the antagonistic roles of Foxn1 and Dkk2 fine-tune HF density and interfollicular spacing during development^142^. Fgf9 and TNFα may in fact mimic Foxn1 as promoters of hair clustering and follicle density. Decades after the discovery of de novo adult hair growth, immune cells have emerged as critical regulators of WIHN encouraging cellular reprogramming through the stimulation of epithelial and dermal developmental programs. To identify the origin and mechanism by which de novo bulge HFSCs and inductive dermal papilla are formed, future lineage tracing experiments must be performed while manipulating the immune niche and examining the impact on cellular dynamics and folliculogenesis.

### Dermal connections

**Fig. 6:**
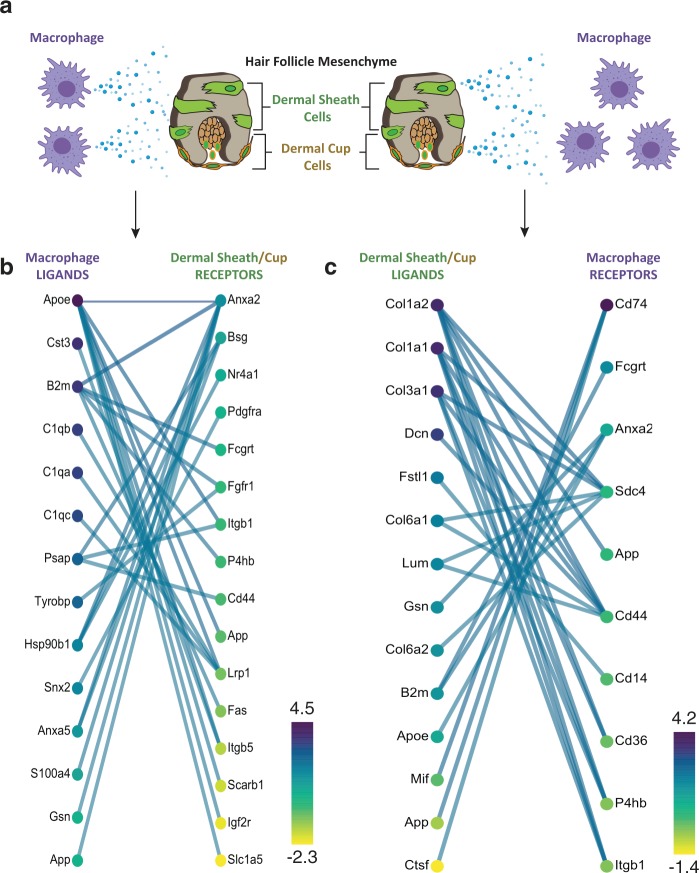
Putative macrophage to HF mesenchyme interactome during murine anagen at post-natal day 28. **a** Schematic illustrating the postulated bidirectional interactome between CD68^+^F4/80^+^ macrophage and CD200^+^/Stmn2^+^ dermal sheath cells and dermal stem cells of the dermal cup during anagen. **b** Unbiased analysis of top predicted interactions (filtered by top 30 weighted edges) using the Cell-Cell Interactions (CCInx) R package revealed immune ligands for which dermal sheath cells have transcriptionally expressed receptors. **c** Unbiased analysis of top predicted interactions revealed HF mesenchyme-derived ligands for which macrophages have transcriptionally expressed receptors. Scale indicates mean normalized gene expression specific to that cell type from our single-cell transcriptomics study of post-natal day 28 anagen skin.

So far, immune-mediated hair regeneration appears to be temporally restricted to telogen and the initiation of anagen with molecular manipulation exclusively targeting epithelial HFSC behavior. Crosstalk between immune cells and other HFSCs at different stages of the hair cycle remains unknown. Dermal stem cells of the dermal sheath are another group of HFSC’s critical for regulating hair regeneration. Is it possible that a set of common ‘core immune signals’ concomitantly activate epithelial and dermal progenitors to produce a new fiber? Moreover, given that anagen maintenance is heterogenous (varying in duration, anatomical location, and response to pathological stimuli), it is possible that immune-derived ligands establish signaling thresholds that extend beyond the telogen–anagen transition and enact an activated state for sustained regeneration. We surmised that an unbiased reassessment of our unpublished single-cell RNA-Seq data where CD200^+^Stmn2^+^ dermal sheath cells (including dermal stem cells of the dermal cup) and CD68^+^F480^+^ peri-follicular macrophages were co-isolated from 28 day old anagen backskin using the 10× Chromium platform would unearth interactions between these two cell types. We utilized the Cell-Cell Interactions (CCInx) R package to generate intercellular communication interactomes by querying Bader Lab’s Ligand-Receptor database^143^. With this method, we identified a battery of macrophage-derived ligands (e.g., C1qa, C1qb, C1qc, ApoE, and Gelsolin) with corresponding receptors on dermal sheath cells (Fig. 6b). We also found multiple macrophage receptors predicted to be activated by various dermal sheath-derived ligands the majority of which appear to be components of the extracellular matrix (Fig. 6c). The extent to which these predicted interactions sustain anagen competency remains to be determined. Elucidating immune signals that interacts with multiple HF stem cells can provide an impetus towards developing maintenance therapies that sustain anagen following initial stimulation.

### Immuned-mediated alopecia: when friends turn to foes

The relationship between HFs and the immune system is defined by one of mutual dependence maintained by the mechanisms of immune privilege (IP). These include diminished MHC class 1 expression in the anagen epithelium making self-antigen presentation challenging, secretion of immunosuppressants (TGFβ1, TGFβ3, adrenocorticotropic hormone, and α-melanocyte stimulating hormone) to create an immunoinhibitory milieu, and the downregulation of T cell activation and proliferation^30,144,145^. The collapse of these immunoinhibitory mechanisms is implicated in the development of immune-mediated alopecias including primary cicatricial alopecias (PCAs) and alopecia areata (AA)^35,37^. What follows is a brief summary of the various immune-mediated alopecias, how dysregulation of the immune-stem cell relationship contributes to their pathophysiology, and what the current therapeutic landscape looks like.

A recent study uncovered a fascinating relationship between stem cell quiescence, antigen presentation and immune evasion unique to bulge and muscle stem cells. Lgr5^+^ bulge HFSCs evade T cell attack and immune clearance through the downregulation of antigen presentation machinery including MHC class 1 and NLRC5 during quiesence^32^. Could failure of this protective mechanism be attributed to the pathogenesis of PCAs such as lichen planopilaris, frontal fibrosing alopecia, and lupus erythematosus? PCAs are scarring alopecias typified by distal perifollicular lymphocytic infiltrates, progressive follicular fibrosis with affected HFs permanently replaced by fibrous tissue^38^. Indeed, biopsies from patients with scarring alopecias revealed IP collapse in bulge HFSCs as evidenced by increased expression of MHC class I and II, decreased expression of TGFβ2, CD200, CD34, and K15, and increased proliferation and apoptosis of bulge HFSCs^146,147^. In contrast, non-scarring forms of alopecia like AA present with IP collapse and immune infiltration along the proximal anagen bulb sparing the bulge. AA is defined clinically by reversible, patchy, and well-circumscribed bald lesions typically on the scalp and beard with dystrophic hairs and a decreased proportion of anagen follicles, a result of their rapid progression into catagen and telogen^35,148^. Histopathology reveals dense immune infiltration of CD8^+^CD3^+^ cytotoxic T cells at the terminal anagen hair bulbs in human and mouse models of AA^149,150^. Considering pigmented hair is more often targeted than nonpigmented hairs, a hypothesized target of cytotoxic T cells has been melanocytes and melanogenesis-associated autoantigens^151^. Genome-wide association studies have implicated a genetic basis for the disease identifying over 100 single nucleotide polymorphisms associated with AA^149^. The genomic regions involved the innate and adaptive immune system controlling, for example, the activation and proliferation of T_reg_ cells, IFNγ response, and activating ligands of the natural killer cell receptor NKG2D. Cytotoxic CD8+NKG2D+ T cells are necessary and sufficient for disease induction in mouse models^34^.

Given the dramatic effect of alopecia on patients’ psychiatric well-being and quality of life, understanding the relationship between HFs and the immune system is vital for the rational development of drugs that restore HF IP. First-line therapeutic options for most immune-mediated alopecias are immunosuppressive agents consisting of either topical, intralesional or systemic glucocorticoid therapy^148,152^. Second and third line agents for managing PCAs include antimalarial agents, dapsone, and finasteride while topical sensitization with ﻿diphenylcyclopropenone and minoxidil have proven to be efficacious in AA management^148,152^. Unfortunately, treating immune-mediated alopecia remains challenging given their chronic clinical course, high rates of spontaneous remission, and relatively obscure mechanism of pathogenesis. In fact, the primary treatment goal PCA is symptom control and slowing down the progression of scarring since hair regrowth is rarely achieved. Immunosuppressive therapies have shown clinical promise as they dampen inflammation through targeted approaches. Pharmacologic inhibitors of JAK protein kinases, downstream effectors of the IFN signature response, have proven to be the most promising. A 2017 retrospective cohort study showed that 77% of patients with AA showed clinical response to tofacitinib^153^. A case series of ten patients with refractory lichen planopilaris showed JAK inhibition with oral tofacitinib to be a promising treatment strategy for PCAs^154^. Low-dose subcutaneous IL-2 also yielded partial hair regrowth and enhanced T_reg_ cell response in 4 out of 5 patients with AA^155^. Oddly, patients receiving anti-TNFα blockers for various autoimmune diseases have been reported to paradoxically develop AA even though TNFα is a cytokine well-known to contribute to the pathogenesis of AA^35,156^. A recent open-label phase 1 and 2 clinical trial has inspired hope and expanded the immune cell and stem cell cooperation paradigm into the therapeutic domain^157^. Nine AA patients with varying disease severity had their mononuclear cells separated in a closed-loop system and “re-educated” after brief contact with adherent human cord-blood derived stem cells before returning to the patient’s blood. The study was able to show a significant and sustained improvement in hair growth and quality of life in AA patients.

The study of immune-mediated hair regeneration sits at the crossroad between basic science research and clinical medicine. Unfortunately, many of the molecular mechanisms driving this mutual relationship have yet to be verified in humans. For there to be any translational success, a concerted effort is needed amongst trichoimmunologists to expand the field’s findings in human disease.

## Conclusions

The cyclical non-scarring regenerative behavior of HFs stands as a vestige of the evolutionary past characterized by an impressive regenerative potential orchestrated by a complex immune milieu. Under homeostatic conditions, the synchronized fluctuation of immune cells ensures an environment conducive for cyclical regeneration by controlling epithelial stem cell quiescence and activation. Injury-induced regeneration, on the other hand, is dictated by immune cells who’s modus operandi is to induce hair regeneration by activating HFSC differentiation programs. HFs secrete chemotactic signals to recruit immune cells during the neonatal period and early phases of wound healing when the cutaneous immune niche requires restructuring. Unfortunately, the human corollary to many of these observations remain elusive. A thorough understanding of the similarities and differences between murine and human immune-mediated regeneration will help develop more effective therapeutics for debilitating immune-mediated alopecias. Trichoimmunology, once an obscure sub-discipline of skin biology, is emerging as a fertile ground for uncovering key principles of immune-mediated regeneration. Next generation sequencing strategies including single cell RNA analysis offer great promise for the field of trichoimmunology.

## Methods

### Single-cell RNA-Seq library construction

All procedures received prior approval from the University of Calgary Health Sciences Animal Care Committee and were completed in accordance with the Canadian Council of Animal Care guidelines (Protocol AC-140019). Single cells were isolated and pooled from *N* = 3 female *Hic1*^*CreERT2*^*:Rosa*^*tdTomato*^ (C57BL/6J background, treated with tamoxifen at postnatal days 3 and 4) mice at postnatal day 28 as previously described^158^. Briefly, anagen backskin was dissected and enzymatically dissociated with dispase for 20 min at 37 °C to remove the epidermis. Remaining dermis was dissociated using 0.2% collagenase for 2 h at 37 °C. Cell suspension was diluted with cold Hank’s Buffered Salt Solution (HBSS), strained through 40 µm cell filters, and centrifuged at 280 × *g*. Liberated single cells were re-suspended in 0.5% bovine serum album in HBSS and partitioned into Gel Bead-In-EMulsions (GEMs) using Chromium Single Cell 3’ Reagent version 2 kit and Chromium Controller (10x Genomics). This process lysed cells and enabled barcoded reverse transcription of RNA, generating full-length cDNA from poly-adenylated mRNA. DynaBeads® MyOneTM Silane magnetic beads were used to remove leftover biochemical reagents, then cDNA was amplified by PCR over 10 cycles. Quality control size gating was used to select cDNA amplicon size prior to library construction. Read 1 primer sequences were added to cDNA during GEM incubation. P5 primers, P7 primers, i7 sample index, and Read 2 primer sequences were added during library construction. Quality control and cDNA quantification was performed using Agilent High Sensitivity DNA Kit. Shallow sequencing was first performed using Illumina MiSeq SR50 to approximate the number of recovered cells. We recovered 3293 single cells with an estimated doublet rate of ≈3%. Based on this, we determined lane distributions for high-depth sequencing using Illumina HiSeq 4000 PE (75 bp paired-end reads) with a targeted coverage of ~115,000 reads per cell.

### Bioinformatics analysis of single-cell RNA-Seq data

Raw reads from Illumina sequencing were processed using 10X Genomics’ Cell Ranger 2.1.0 pipeline with default and recommended parameters^159^. FASTQs generated were aligned using STAR aligner to a custom GRCm38.p5 mouse reference genome. Next, feature-barcode matrix containing unique molecular identifier (UMI) counts were imported into Seurat V3 R toolkit where low quality cells (<200 or >3000 genes/cell, <20,000 UMIs) were excluded from analysis^160^. Gene expression was log normalized to a scale factor of 10,000 and unsupervised clustering of all cells was performed. Seurat’s *FindAllMarkers* function (with “negbinom” test) was used to identify differentially expressed genes and clusters were annotated based on previously established transcriptional signatures. *Pdgfra*^+^ fibroblast cluster co-expressing *Cd200* and *Stmn2* was annotated as ‘hair follicle dermal sheath cells’ and *Cd45*^+^ immune cluster co-expressing *Cd68* and *F480* (but negative for *Cd207*, a marker of resident Langerhans) as ‘peri-follicular macrophages’^161–163^. Cell-cell interactions between hair follicle dermal sheath cells and peri-follicular macrophages were predicted using the *BuildCCInx* function (mmusculus used as the data source) in CCInx R tool which restricts analysis to genes present in the Bader Lab ligand-receptor interaction database^164^. Bipartite graphs presented were generated using an interactive Shiny application launched with *ViewCCInx* function.

### Reporting summary

Further information on research design is available in the Nature Research Reporting Summary linked to this article.

## Supplementary information

Reporting Summary

## Data Availability

The single-cell RNA-seq sample reported in this study has been deposited in NCBI Gene Expression Omnibus (GEO) under the accession number GSM2910020. Raw data is available in Sequence Read Archive (SRA) under the accession number SRX3581877.
